# Supplementary material for: Are carbon emissions trading and green financial instruments synergistic? -Comprehensive quantitative research based on content analysis
Source: PLoS One. 2024 Mar 7;19(3):e0298601. doi: 10.1371/journal.pone.0298601 (PMC10919601; doi:10.1371/journal.pone.0298601)
Supplement: S1 Data — (ZIP) [file pone.0298601.s001.zip › Data/地级市Z_F工作报告.zip!/╝¬┴╓/╦╔╘¡╩╨/└┤╫╘╣1⁄2╓┌║┼í╛┬φ┐╦╩2╛▌═°í┐_2017.docx]

各位代表：

　　现在，我代表市人民政府，向大会作工作报告，请予审议，并请市政协各位委员提出意见。

　　一、2016年工作总结及本届政府工作回顾

　　刚刚过去的2016年，是松原发展进程中极不平凡的一年。这一年，我们在省委、省政府和市委的正确领导下，团结带领全市各族人民，深入贯彻落实习近平总书记系列重要讲话精神，牢牢把握吉林省西部生态经济区建设这个统领，紧紧围绕建设绿色产业城市和生态宜居城市，坚决打好稳增长保卫战、调结构攻坚战、促发展持久战，在攻坚克难中奋力拼搏，在改革创新中抢抓机遇，在真抓实干中开拓进取，较好地完成了各项工作目标任务。

　　一年来，我们科学施策、砥砺前行，经济发展稳中向好。预计（下同）全市地区生产总值实现1800亿元，同比（下同）增长6.5%；地方级财政收入完成50.7亿元，增长1%；全社会固定资产投资完成1480亿元，增长13%；社会消费品零售总额完成665亿元，增长10%；外贸进出口总额实现1.7亿美元，增长19%；城镇和农村常住居民人均可支配收入分别达到30830元和10087元，分别增长6.5%和5.5%。

　　工业提速转型。新增规模以上工业企业20户；规模以上工业增加值实现690亿元，增长5.3%。非油工业增加值和税收分别达到545亿元、11.5亿元，分别增长10%和5.5%。主导产业稳中有进。油气产量当量完成496万吨，松原石化10万吨丙烯和众诚油页岩原位裂解中试基地项目扎实推进，众诚油页岩松原院士工作站挂牌成立，天伦合成焊割气项目投入生产，金海5500吨化工助剂项目开工建设。查干湖大米产业园投产达效，中粮集团饲料厂投入试生产，天船马铃薯全产业链和国鑫冷藏农产品加工项目主体完工，交投10万吨玉米全籽粒高值化综合开发项目生产车间封顶，盼盼食品产业园项目二期开工建设。农产品加工业增加值首次超过油气开采业，成为第一主导产业。优势产业好中提升。史丹利60万吨复合肥、中盐红四方80万吨复合肥一期、新洋丰80万吨新型复合肥等项目竣工投产。新研牧神农机项目二期完成设备安装，澔泰专用车制造和天德玻璃钢管项目开工建设。新兴产业变中突破。达肺草口服液项目投入试生产。阳光凯迪生物质合成油战略示范基地和天润风电制氢国家级示范项目完成桩基工程。中科惠锋光电材料产业园项目完成规划设计。北京SLEC冷光源、上海超导二代高温带材、江西德义半导体等一批高精尖项目顺利推进。

　　农业提质增效。一产增加值实现310亿元，增长5.7%。农业生产能力稳步提升。哈达山水利枢纽工程发挥综合效益，松原灌区工程顺利推进，引松入扶一期工程竣工通水，川头尾闸、阎家围子连接渠等河湖连通应急工程完工并发挥补水功能。新建高标准农田41.6万亩。主要农作物综合机械化作业水平达到85%。积极开展人工影响天气作业，战胜严重旱灾虫害，粮食总产量达到740万吨。现代农业发展步伐加快。市农科院完成组建。16个绿色农业示范基地建设成果显著。海峡两岸（吉林）生态农业合作先行实验区获批建立，“善德良米”有机大米成为杭州G20峰会指定用米。本然生物土壤调理剂试验示范成效明显。调减普通玉米种植面积79万亩，有机绿色无公害作物种植面积达到190万亩，种植中药材4000亩。新增农民专业合作社、家庭农场和专业大户2600户。流转土地293万亩，土地规模化经营面积达到375万亩。精品畜牧业蓬勃发展。华西希望生猪养殖一期、正邦集团200万头生猪养殖加工一体化二期、乾羊肉羊屠宰精深加工一期、科尔沁万头绿色肉牛养殖等项目建成投产，大北农60万头生猪养殖项目开工建设。新建标准化规模养殖场（小区）170个。无抗养殖技术有效推广。畜牧业增加值实现95亿元，增长5.2%。

　　服务业提档升级。全力打好服务业发展攻坚战，服务业增加值实现720亿元，增长8.8%。突出发展特色旅游业。成功举办首届查干湖莲花文化旅游节；查干湖冰雪渔猎文化旅游节跻身“2016最美中国榜”，荣膺“全国首批最具影响力特色节庆”称号。天河谷温泉小镇温泉馆项目完成一期主体工程，华西希望“花舞松原”和威尼斯水上乐园项目开工建设。旅游综合收入增长17%，突破百亿元大关。加快发展商贸物流业。镜湖半岛商贸体建成运营，大润发超市江北店完成征地拆迁，永正国际沃尔玛超市项目顺利推进。瑞禾仓储物流园跨入省级现代服务业集聚区行列，江苏敬亿国际商贸物流城项目完成前期工作，现代空港物流产业园项目签约落地，邮政快递业务发展迅猛。稳步发展房地产业。完成投资25亿元，开发房地产76万平方米；销售商品房75万平方米，增长7.5%。中信城、润阳国际等地产项目相继建成。积极发展金融业。存贷款余额分别增长14%和15.3%，贷款增量创百亿元新高。光大银行松原分行获批设立。中华联合财险公司在我市设立分支机构。阳光村镇银行全国农业金融服务暨松原市电子商务中心投入运营。延边农商行松原分行、扶余惠民和长岭蛟银村镇银行开业运营，村镇银行实现县域全覆盖。大力发展信息服务业。深入落实“互联网+”行动计划，“爱松原”工程正式启动，供销宝电子商务平台成功上线，北京鼎九互联网服务、农业大数据示范工程、金融大数据中心和量子通信产业园项目稳步推进。

　　一年来，我们内引外联、合力攻坚，招商引资和项目建设取得突破。招商引资成果丰硕。松原商业联合会正式成立。市招商服务公司完成组建。积极参加首届全球吉商大会、世界500强企业走进吉林、台企吉林行、全省第九届海外华侨华人专业人士恳谈及项目对接会等招商活动，签约正大集团300万只蛋鸡全产业链和120万吨稻米深加工等重大项目14个。成功举办海峡两岸经营俱乐部高峰论坛、全国杂粮峰会、中国粮油财富论坛和“生态文明、绿色发展”等高端战略研讨会。赴北京、上海、成都、武汉、南京等地进行高密度、高频次沟通对接，赴美国、以色列开展经贸交流活动，与盈创科技、福创科技和嘉吉集团等企业的战略合作取得积极成果。招商引资到位资金增长12%。项目建设扎实推进。重大项目建设“百日会战”成效显著。开复工5000万元以上项目271个；完成固定资产投资426亿元，增长11.5%。新谋划5000万元以上重大项目292个。全面加强重大项目入库管理和三年滚动投资计划编制，争取国省专项基金和预算内资金25亿元。

　　一年来，我们突破藩篱、勇涉深水，各领域改革不断深化。着力深化供给侧结构性改革。全面落实“三去一降一补”任务，积极消化水泥、风电过剩产能，努力去除住房、玉米等商品库存，大力推进房地产企业、中小企业去杠杆，供给侧结构性改革取得重要阶段性成果。着力深化金融改革。农村金融综合改革试点稳步推进。成功组建松原城市发展投资控股集团、现代农业投资控股集团和生态文化旅游产业投资控股集团，累计完成融资100亿元，为全市经济转型、城市建设、民生改善等提供了强有力支撑。吉林和元生物公司在全国中小企业股份转让系统挂牌，我市中小企业在“新三板”上市实现零突破。着力深化农业农村改革。农村土地确权登记颁证、集体资产产权登记、耕地质量评定与保护、扩权强镇等改革试点扎实开展，农村土地承包经营权抵押贷款业务全面铺开，农村棚膜设施确权颁证试点工作扎实推进，秸秆收储公司、棚膜经济投资公司和养殖场建设投资公司完成设立，供销合作社和国有林场改革启动实施。此外，吉林油田公司“四供一业”分离移交完成资产核查和供水、供热单栓改造等前期基础工作。基层医疗卫生机构综合改革不断深化，我市被确定为全国第四批城市公立医院综合改革试点市。国有企业、公务用车、公交车经营等改革有序推进，机关事业单位养老保险制度、市直机关事务管理体制等改革进展顺利。

　　一年来，我们紧扣生态、突出宜居，城乡建设统筹推进。中心城市建设呈现新风貌。《滨江生态控制性概念规划》和海绵城市、城市燃气、轨道交通等19个专项规划编制高标准完成。新城东路、建设街、康宁街等9条街路新建续建工程全面竣工。建成学府街、文化路等路段地下综合管廊。五环大街、伯都讷大街等19处市政工程和公园、广场全面完成绿化彩化。天河大桥桥体和东镇大路、滨江大道等街路及周边楼体亮化全面升级。圣湖补水工程完成九曲湖生态补水。广大居民企盼多年的无名泄支水环境综合整治工程正式启动。江北净水厂哈达山取水工程总量完成过半。生活垃圾焚烧发电及江南、江北污水深度处理等项目投入运行。县城和美丽乡村建设迈出新步伐。扶余、前郭、长岭、乾安的城市面貌进一步改观。三井子、查干花等10个示范镇启动建设。55个省县级“示范村屯”完成绿化美化任务。“厕所革命”扎实推进，改造农村厕所7500个。新建农村公路375公里。立体化交通建设开启新阶段。查干湖机场建设实现暖封闭，宁江客运站主体工程完工，铁科高速、长白快铁、长西铁路松原段和客运交通枢纽与站前综合改造等重大交通项目建设进展顺利。

　　一年来，我们心系百姓、改善民生，幸福松原建设全面推进。突出实施民生行动计划。认真兑现民生承诺，全力办好31件民生实事。新增城镇就业4.5万人，转移农村剩余劳动力70万人次。发放创业担保贷款1.1亿元。养老、失业、工伤、生育等社会保险进一步扩面。全面完成城乡低保提标。积极开展“情暖松原·红十字博爱圆梦”捐助贫困大学生活动。发放救灾救济资金232万元。投放住房公积金贷款7.7亿元。改造棚户区和城区危房55万平方米、农村危房8113户，建设购买安置住房5671套，分配廉租房和公租房650套。完成油田矿区“暖房子”工程169万平方米。新建、改造中心城区背街巷路6条。市城区集中供热联网面积达到2900万平方米。全程检测哈达山水库等饮用水源，扎实推进农村饮水安全巩固提升工程，城乡居民饮水安全得到有效保障。市食品药品检验中心完成桩基工程。全面打响脱贫攻坚战。累计投入资金13.6亿元，大力发展高效农业、庭院经济和劳务经济，实施各类产业项目116个；修建重点村屯道路640公里，改造危房4573户，铺设低压线路340公里，完成1.2万户安全饮水改造项目；积极改善薄弱学校办学条件；帮助贫困群众缴纳新农合和大病保险资金；有针对性开展各类技能培训活动，切实增强群众脱贫致富本领。62个贫困村实现脱贫摘帽，21366户44185名贫困群众告别贫困。大力加强生态环境治理。单位地区生产总值能耗下降3个百分点。全面完成市供热公司脱硫脱硝改造项目，淘汰市城区10吨以下燃煤小锅炉76台，城区换热站和棚户区燃煤能源替代积极推进，治理加油加气站279座，淘汰黄标车和老旧机动车1.9万辆，严控秸秆露天焚烧，城乡空气质量明显改善。查干湖生态环境保护试点工程完成年度建设任务，松花江、嫩江干流综合治理工程启动实施。全面发展社会事业。市教育公共服务云平台工程一期建成使用，市钻井小学、运输小学等学校教学楼和市实验高中全民健身中心完成建设。二氧化碳驱动采油等4项科技成果荣获省级科技进步奖，市清华科技园被认定为省级科技企业孵化器，民乐蔬菜等4个特色种植养殖基地被科技部认定为国家级星创天地。市中心医院易地新建项目完成主体工程，扶余市中医院项目完成土建工程，编制发放新版《松原居民健康知识手册》6万册。困难残疾人生活补贴和重度残疾人护理补贴制度全面启动。松原文化传媒公司组建成立，成功举办市民文化节、农民文化节系列文化活动。《松原年鉴（2016）》完成编纂。广播电视无线数字化覆盖工程建成使用。圆满承办查干湖杯全国桥牌邀请赛等重要赛事，我市运动员在全国冬运会上取得优异成绩，市体育馆全面竣工并即将对社会开放，市城区25处健身场地体育器材配建项目全面完工。市档案馆建设项目启动实施。市防灾减灾指挥中心完成土建工程。

　　一年来，我们创新治理、从严治政，民主法制和政府建设不断强化。着力加强社会治理。七五普法扎实开展。圆满完成第十届村民委员会换届选举。突出解决群众反映强烈的热点难点问题，化解信访积案113件。“打非治违”专项行动成效显著，重特大安全事故得到有效遏制，妥善处置突发事件13起。深入开展“打盗骗、保民安”等专项行动，禁毒攻坚战取得重大胜利，网络管控能力进一步增强，公安武警武装联勤实现常态化，群众的安全感和满意度进一步提升。着力加强政府自身建设。坚持“法定职责必须为、法无授权不可为”，深入推进依法行政。自觉接受市人大及其常委会法律监督、工作监督和市政协民主监督，办理市人大代表建议、政协提案206件。扎实开展“两学一做”学习教育和政府机关作风建设年活动，全力推进政务公开和政务服务“一张网”建设，持续深化“放管服”改革，进一步强化行政监察和审计监督，公职人员服务意识和机关行政效能进一步增强。

　　各位代表！2017年，市政府按照要求提前换届。回顾本届政府任期的四年，是我们在宏观经济环境趋紧的大背景下，遭受原油量价齐跌严重冲击和地震、洪涝、干旱等重大自然灾害严峻考验的四年；是松原在建市20周年的新起点上，经济转型发展、“两个城市”建设迈出坚实步伐的四年；也是我市城乡面貌变化更大、人民群众受益更多的四年。四年的奋力拼搏，我们在全面建成小康社会的征程上，写下了厚重的一页。四年来，全市综合实力迈上新台阶。本届政府任期末，全市地区生产总值比2012年净增195亿元，经济总量稳居全省第三位并不断壮大；地方级财政收入克服涉油收入年均下降28%等不可控因素影响，成功遏制负增长态势；全社会固定资产投资是2012年的1.4倍，连续4年保持千亿级投资强度；社会消费品零售总额是2012年的1.4倍，保持年均两位数高速增长；外贸进出口总额是2012年的1.3倍，增速实现全省“两连冠”。四年来，争创中国驰名商标、国家地理标志商标和吉林省著名商标118件，培育创建吉林省名牌产品43个；我市被国务院列为全国唯一的生物质能源产业集群城市和全省唯一的成长型资源城市，成功跻身“中国最具幸福感百强城市”、“全国文明城提名城市”，荣获“全国优秀民生改善典范城市”、“全国人民防空先进单位”、“省级双拥模范城”称号和“中国扶贫·政府创新奖”、“吉林省质量管理奖”，松原的知名度和影响力与日俱增。产业结构调整实现新突破。强力推动新型工业、现代农业、现代服务业加快发展，三次产业比重调整到17.2︰42.8︰40。经开区跨入国家级开发区行列。盼盼食品工业园、中粮健康生猪产业化、乾源50万立方米LNG、苏源50万吨复合肥等一批重大项目投产达效，石油开采和化工、食品和农产品加工、装备制造、化肥等多元支撑的工业格局初步形成。建设国省级农业标准化示范区11个，粮食生产能力达到750万吨阶段性水平，农产品交易总量突破300万吨，畜牧业增加值占一产比重突破30%，扶余市、长岭县分别发展成为全国最大的花生生产基地、全国闻名的葵花和马铃薯生产基地。新型服务业态发展迅速，服务业增加值占GDP比重逐年提高。美丽家园焕发新容颜。中心城区建成区面积达到67平方公里。江南江北、地上地下建设统筹推进，累计投资420亿元，建设重大城市基础设施项目45个。壅水坝、天河大桥等一批标志性工程和供排水、供热、燃气等一批重点市政工程相继建成使用，城市综合承载能力显著提升。“一江两岸”成为中心城区最亮丽的风景，城市的秀美宜居度和形象品位大幅提升。哈大客专松原段、松陶铁路、绕城高速建成通车，立体化交通体系初步形成。全市城镇化率达到44.8%。人民生活得到新改善。城镇和农村常住居民人均可支配收入分别突破3万元和1万元大关，年均增速均跑赢同期GDP增速。连续四年组织实施民生行动计划，累计投资204亿元，建设重点民生项目261个，全市百姓获得更多“暖心”实惠。成功夺取抗震救灾和灾后重建全面胜利，创造了连续强震下“无一人死亡、无一人重伤”和“一年受灾、半年恢复”的奇迹，地震灾区建成温馨家园。回顾本届政府任期的四年，困难超出预期，成绩来之不易。经过四年的攻坚克难、开拓奋进，松原已经站在了转型发展的更高起点，迈入了振兴发展的崭新阶段。

　　各位代表！过去四年，我们在继承中创新，在发展中突破，在探索中前行。四年实践，我们深切地感受到：必须坚持真抓实干。坚定不移地贯彻落实党中央国务院、省委省政府和市委的决策部署，不断探求符合时代特点、发展特征、松原特色的发展模式，以担当有为的精神践行发展战略，以抓铁有痕的作风增强发展动力，以夙兴夜寐的干劲开创发展新局。必须坚持转型发展。坚定不移地走有质量、可持续的发展道路，审时度势、未雨绸缪，科学设计、大胆开拓，扎实推进转型升级、创新驱动，积极发展战略性新兴产业，以规模扩张促进稳定增长，以质量提升增强发展后劲，强力推动松原发展蓄势扬帆、行稳致远。必须坚持深化改革。坚定不移地以深化改革破难题、促发展，坚持“放管服”改革的总体方向，既充分发挥市场内在牵引、利益驱动作用，又充分发挥政府导向引领、定向调控职能，形成有效市场、有为政府的有机统一，使资源配置更加高效、经济发展更具活力。必须坚持执政为民。坚定不移地把保障和改善民生作为一切工作的出发点和落脚点，时刻把老百姓的利益放在心上、抓在手上、扛在肩上，带着感情、带着责任、带着使命，真心实意地为群众做好事、办实事、解难事，全力寻求解决群众诉求最大“公约数”，努力把发展蓝图变成增进群众认同感、获得感、幸福感的生动现实。

　　各位代表！四年来成绩的取得，是省委、省政府和市委正确领导、统揽全局的结果；是市人大和市政协有效监督、大力支持的结果；是在历届政府和老领导、老同志打下的坚实基础上，全市广大干部群众团结拼搏、锐意进取的结果；是中省直单位、驻松部队、武警官兵、消防战士和社会各界热情参与、携手共建的结果。在此，我代表市人民政府，向所有为松原发展建设作出贡献的同志们、朋友们，致以崇高的敬意和衷心的感谢！

　　各位代表！在看到成绩的同时，我们也清醒地认识到，工作中还存在不少困难和问题，主要表现为：“一油独大”的产业结构和经济格局还没有实现根本转变，新兴产业、民营经济发展缓慢；原油、天然气、粮食等资源优势转化成产业优势的亮点不多；创业创新能力不足，高层次人才短缺，具有核心竞争力的技术十分匮乏；一些改革措施还没有完全落实到位；国有资源、资产、资本管理体制不健全，利用效益不高；民生保障存在短板，脱贫攻坚任务十分繁重；个别干部不作为、慢作为、乱作为的问题仍然不同程度存在，营商环境还需要进一步优化，等等。对此，我们一定高度重视，在今后工作中认真加以解决。

　　 二、今后五年工作思路和2017年主要任务

　　未来五年，是我市推进生态振兴、转型崛起的关键期，是全面建成小康社会、全面建成“两个城市”的决胜期。市六次党代会准确把握中央、省委方针政策和松原市情，科学提出了未来五年我市经济社会发展的指导思想、奋斗目标和主要任务。我们必须紧密结合实际，认真抓好贯彻落实。

　　今后五年政府工作的总体思路是：高举中国特色社会主义伟大旗帜，以邓小平理论、“三个代表”重要思想、科学发展观为指导，深入贯彻习近平总书记系列重要讲话精神，按照中央“五位一体”总体布局、“四个全面”战略布局和省委“三个五”发展战略，抢抓新一轮振兴东北重大机遇，践行“五大发展”理念，坚持生态振兴、转型崛起，坚定不移抓住“一个统领”，聚精会神建设“两个城市”，全力以赴打好“三大战役”，变中求新、变中求进、变中突破，做好“有中生新、无中生有”文章，在引领经济发展新常态中寻求新突破，在推进供给侧结构性改革中积聚新动能，在全面推进社会建设中取得新进展，努力建设经济更繁荣、城乡更优美、民生更普惠、社会更和谐的幸福松原。

　　未来五年，我市经济社会发展的主要预期目标是：地区生产总值年均增长7%左右；地方级财政收入与经济保持同步增长；三次产业比重调整到13∶44∶43，绿色产业增加值比重达到50%以上，高新技术产业增加值占规模以上工业比重提高8.2个百分点；单位地区生产总值能耗、二氧化碳排放总量、主要污染物排放总量持续减少，城市空气质量优良天数逐年增加，森林覆盖率达到11%以上，城镇供水水源地水质达标率实现100%；常住人口城镇化率达到53%；城乡常住居民人均可支配收入年均增长7%左右。

　　展望未来，机遇与挑战并存，希望与困难同在，但是机遇和希望大于挑战和困难。特别是国家实施新一轮振兴东北战略，密集出台一系列含金量高、操作性强的政策措施，更加有利于我们加快推进产业结构调整、加速提高发展质量效益、全面增强发展活力和内生动力；中央实行积极的财政政策和稳健的货币政策，更加有利于我们精准对接国省专项资金和社会资本，更好地满足重大产业、基础设施和民生项目建设的资金需求；中国制造2025、农业现代化、“互联网+”行动计划、打造哈长城市群和吉林省西部生态经济区建设等重要战略机遇，更加有利于我们向上争取政策支持、向内转换支撑动力，加速培植经济发展后劲。我们有信心、有决心、有恒心深入践行“创新、协调、绿色、开放、共享”发展理念，全面完成未来五年各项目标任务，努力开创松原经济社会发展新局面！

　　2017年是新一届政府开局之年，工作的总体要求是：深入贯彻落实中央和全省经济工作会议精神，按照市六次党代会部署，坚持稳中求进工作总基调，适应把握引领经济发展新常态，坚持以提高发展质量和效益为中心，坚持以推进供给侧结构性改革为主线，统筹推进创新驱动、项目带动、资本撬动，全面做好稳增长、调结构、促改革、惠民生、防风险各项工作，促进经济平稳健康发展与社会和谐稳定。全市经济社会发展的主要预期目标是：地区生产总值增长6.5%，地方级财政收入与经济保持同步增长，全社会固定资产投资增长8%，社会消费品零售总额增长10%，城乡常住居民人均可支配收入与经济保持同步增长。新的一年，要突出做好以下重点工作：

　　（一）千方百计兴工业。围绕转型升级和规模扩张，坚持“加减乘除”一起做，突出抓好结构调整和项目攻坚，加快积蓄工业经济坚实增长新动能。努力壮大油气经济。积极支持吉林油田公司、东北油气公司遏制原油减产，新打油井866口，推动中石化长岭201井试采厂平稳运营，争取油气产量当量达到440万吨。促进金海5500吨化工助剂和中科聚合科技聚芳醚酮等项目竣工投产，争取启动吉港50万吨LNG项目，推动摩擦技术应用产业园项目落地。加快发展非油工业。着力发展农产品精深加工业，推动信诚爱谷50万吨绿色蔬菜产业园投入生产，开工建设中欧食品产业园、金字塔现代农业产业园、嘉吉玉米生化产业园和变性淀粉、吉荣50万吨绿豆全籽粒高值化综合利用等项目，加快推进正大集团120万吨稻米深加工等项目。着力发展医药产业，支持百年汉克、华侨制药等企业做大做强，推动尼普洛中硼硅药用包装项目竣工投产。着力发展化肥产业，推动北京谷田30万吨掺混肥和20万吨微生物菌肥项目建成投产、中盐红四方80万吨复合肥项目二期投入试生产。着力发展装备制造业，推动中科惠锋光电材料产业园和新研牧神农机二期项目投产运营，开工建设阳光汽车配件二期、殴彼龙汽车轮胎及防冻液和山东润峰光伏绿色能源产业基地项目，推动江西德义半导体项目落地。着力发展生物技术产业，开工建设味之源大豆生物科技产业基地和生物质材料产业园项目，加快推进阳光凯迪生物质合成油战略示范基地、长白山开发集团80万吨秸秆综合利用、本然生物土壤调理剂母料、京福龙酵之谷等项目。

　　（二）多措并举强农业。努力争作全省农业现代化的“领头羊”，加快构建现代农业生产、经营和产业体系，深入推进农业供给侧结构性改革，精选品种、做优品质、打响品牌，正常年景粮食总产量达到750万吨。突出试验示范。致力于打造全国绿色有机农业示范市和全国知名的绿色农产品基地，绿色农业示范基地发展到30个，加快海峡两岸（吉林）生态农业合作先行实验区和三江口现代农业示范区建设，新增高标准农田35万亩，有机绿色无公害农作物种植面积达到200万亩。加快引进品种改良、精准施肥、节水灌溉、生物防治等先进技术。大力发展瓜果、食用菌等特色种植，棚菜种植面积发展到11.9万亩。依托中医药龙头企业，组建生态中药材开发投资公司，大力建设规范化中药材和种苗种植基地，中药材种植面积达到5500亩。突出设施建设。积极推进松原灌区、河湖连通、引松入扶二期等重点工程建设，做好三江口灌区建设前期工作，新增农机总动力20万千瓦。突出集约经营。大力推广长岭兴源合作社成功经验，农村合作组织和家庭农场等新型农业经营主体发展到3.5万个，土地规模化经营面积达到400万亩。突出精品养殖。大力推广使用无抗养殖技术。新建标准化规模养殖场（小区）170个。加快推进华西希望生猪养殖、正大集团300万只蛋鸡全产业链、特沃斯牧业万头精品肉牛等重大优质项目。

　　（三）换挡升级提三产。紧扣产业关联、业态融合和有效供给，深入挖掘服务业发展潜力，积极培育新的经济增长点。大力发展特色旅游业。深入实施“查干湖+”行动计划，办好查干湖冰雪渔猎文化旅游节、莲花文化旅游节和摄影大赛等系列活动；深化与海航集团、国开金融等企业合作，统筹推进查干湖“五大工程”建设，大力发展生态湿地、冰雪体验、民俗文化、休闲观光农业等特色旅游，全力打造“冬捕经济”、“查干湖经济”，加快发挥查干湖旅游业在生态振兴、转型崛起中的示范带动作用。与携程集团开展旅游全产业链合作，加快建设查干湖等10个特色旅游小镇，推动天河谷温泉小镇项目投入运营、华西希望“花舞松原”世界花海一期工程完工，加快威尼斯水上乐园项目建设进程，开工建设灵山寺景区。大力发展商贸物流业。开工建设大润发超市江北店，积极推进电商大厦和万达城市广场项目。推动瑞禾仓储物流园三期工程投入运营，加快推进江苏敬亿国际商贸物流城建设，开工建设北京新发惠农国际现代农业综合服务园和松陶铁路沈铁物流园。大力发展房地产业。完善规划引领、科学供地机制，促进房地产有序开发，增加普通和中档商品房供应，加大保障性住房建设力度，满足群众自住和改善性住房消费需求，促进房地产业回暖。大力发展健康养生产业。依托生态资源优势，加强与华大基因、台湾晶宇生物芯片等大企业战略合作，谋划建设健康养生产业园，稳步推进中药材收储、中医药健康产品生产项目建设。大力发展金融业。支持光大银行松原分行挂牌运营，做好宁江惠民村镇银行等企业在“新三板”挂牌工作。积极引进信托、基金、融资租赁等新型金融机构。持续深化与国家政策性银行、国有商业银行和重点股份制银行战略合作，广泛争取各类基金投资松原。全市银行业金融机构存贷款余额分别增长12%和14%。大力发展信息产业。全面实施“爱松原”工程，开展电子商务进农村综合试点，推动云计算大数据中心建成运营，加快推进金融大数据中心、量子通信产业园和农业大数据示范工程项目。大力发展文化创意产业。启动春捺钵遗址群保护工程，加快建设市文化创意产业园和前郭鼎润非遗活态传承展览馆，统筹做好查干湖、大布苏泥林和辽金历史、蒙古族民歌等文化创意文章，充分发挥松原文化传媒公司作用，把特色鲜明的文化资源优势转化为充满活力、富有魅力的文化产业优势。

　　（四）统筹城乡美家园。树牢“绿水青山就是金山银山”理念，紧扣生态振兴，加强系统规划、统筹建设、精细管理，全力争取列为国家中小城市PPP模式创新试点，努力打造生态宜居的美丽家园。着力推进“一江两岸”综合开发。深入实施沿江开发战略，启动海绵城市建设一期工程，扎实推进圣湖补水项目，开工建设沿江百里绿廊，完成纳仁汗公园二期建设和扶余公园改造项目，进一步彰显滨江亲水、绿色生态的城市特色。着力推动城市路网建设。完成康宁街、建设街、团结街等道路建设改造。开工建设天河大桥南北岸互通立交工程。完成客运交通枢纽与站前综合改造和宁江客运站项目，实现铁路、公路和城市公共交通“零换乘”。全面推进查干湖机场建设，确保年内建成通航，打通连接环渤海、长三角和珠三角等地区的“空中走廊”。实现长白快铁项目竣工并开通松原始发列车，加快建设长西铁路松原段。启动实施查干湖至机场高速项目，积极推进长太高速松原段项目建设，完成铁科高速征地拆迁等前期工作。着力加快地下综合管廊建设。实施文化路、学府街、环保大街、伯都讷大街等20公里路段管廊建设，形成廊体10公里；完成过江隧道综合管廊前期工作及PPP项目公司组建，争取年底前启动项目建设。着力实施无名泄支水环境综合整治工程。致力于打造南部城区发展的生态带、景致秀丽的水走廊、沿线百姓的幸福渠，启动实施城区29.7公里无名泄支水环境综合整治，完成河道疏浚等工程，同步启动周边棚户区改造和公共设施建设。着力改善群众居住条件。深入开展“一拆三改”行动，实现“三个区域”全覆盖，完成房屋征拆和土地征收200万平方米。改造棚户区20.8万平方米、农村危房7600户，综合整治老旧小区20万平方米，完成松苑雅居小区廉租房分配任务。办理1.7万户160万平方米无籍房不动产权登记颁照，解决困扰群众多年的房屋产权流转“老大难”问题。进一步理顺城区供热体制，改善城区供热条件，优化城区热源布局。着力提高城市管理水平。继续保持严管态势，深入开展城区物业、市容环境和城市出口综合整治。加强道路交通治理。积极推进智能化城市管理信息指挥中心建设，进一步提升城市智能化、规范化、精细化管理水平，让整洁舒适成为广大市民的“生活福利”。着力加强县镇村建设。稳步推进各具特色的县（市）城建设，积极支持扶余打造成为哈长城市群重要节点城市、长岭打造成为特色区域小城市、乾安打造成为生态百湖城。加大乌兰图嘎、太平川等特色镇建设投入力度。开工建设乡镇生活垃圾收转运体系项目，持续抓好49个省级重点村建设，新建“美丽庭院”2140户、“干净人家”6040户，改造农村厕所3000个，新建农村公路150公里，加快建设一批别致多样、“留得住乡愁”的美丽乡村。着力强化生态文明建设。贯彻落实国家大气、水、土壤污染防治行动计划，加快实施蓝天、碧水、绿地三大工程，实行大气污染治理“五控”并举，统筹推进松花江、嫩江干流综合治理工程，启动建设查干湖人工湿地和引水渠，推动龙凤湖和腰井子羊草草原自然保护区晋升为国家级自然保护区，高标准绿化松原大地，让生态更好地造福全市人民。

　　（五）内改外联增动力。深化重点领域改革，吸引域外战略投资，不断凝聚发展正能量、培育发展新动能。扎实推进行政管理体制改革。完善城市管理领域机构综合设置。完成市直机关事务管理体制和公务用车制度改革。进一步理顺市本级教育行政管理体制。整合建立统一的公共资源交易平台，筹划设立市公共资源交易中心。扎实推进国有企业改革。完善国有资产监督管理体制，健全松原城市发展投资控股集团、现代农业投资控股集团和生态文化旅游产业投资控股集团等市属国有企业法人治理结构，落实国有资产保值增值责任。加快解决历史遗留问题，盘活国有资产存量，提高国有资产运营效益。完成吉林油田公司“四供一业”资产移交，启动供水、供热等设施改造。扎实推进农业农村改革。全面完成土地承包经营权确权登记颁证。建立农村土地流转交易平台。统筹做好秸秆资源收储、开发和利用。深入开展“粮食银行”试点工作。积极推进国有林场和供销社改革。扎实推进金融等领域改革。深入开展农村金融综合改革试验，创新林地、草场、水面等资源性物权融资产品，成立市农村物权融资登记托管交易公司。加快推进农村棚膜设施确权颁证，支持发展抵押融资。推动宁江、扶余、乾安、长岭农信社改制为农商行。加快推进城市公立医院、机关事业单位养老保险制度等方面改革。扎实推进民营经济发展。以“大众创业、万众创新”为载体，重点支持“专精特新”中小企业、“质量标杆”企业、科技型中小企业加快发展。深入推进正税清费、降本减负，加快打造中小企业公共服务、市场拓展和共性技术三大平台，完善市“三押”融资服务中心服务功能。建立现代企业制度试点，健全完善民营企业信用评价服务体系。全力申报国家东北地区民营经济改革试点城市，民营经济主营业务收入增长6%。立足转型谋项目。围绕我市产业基础、产业走向、资源禀赋和“四高”要求，锁定国家重大工程包，积极邀请光机所、应化所、吉林大学等重点科研院所、国内知名高校参与项目谋划、评估和论证，切实把项目谋实谋深谋精，提高储备项目的成熟度。立足创新引投资。锁定“北上广”，瞄准“高精尖”，紧盯“中字号”、“国字头”和上市企业，深化与中海油集团等企业的战略合作，着力引进科技前沿、市场领先、能够支撑和助推全市经济发展的大型领军企业。全年引进亿元以上大项目60个，招商引资到位资金增长12%。立足聚商建机制。支持松原商业联合会发起设立松商投资集团。用好政府引导基金，完善奖惩并重的招商考核机制，全面落实县（市区）、开发区招商引资主体责任，开展项目对促进经济增长、财税增收、增加就业、集约用地、改善生态环境的综合评估，推动招商引资取得更加丰硕的成果。

　　（六）贴近百姓惠民生。大力实施民生行动计划，扎实做好基础性、兜底性、普惠性民生工作，全力打造幸福松原升级版，让城乡居民生活得更加美好。努力扩大社会就业。大力开展劳务输出对接、劳动力转移培训和城乡贫困妇女实用技能培训，启动农村就业制度综合改革试点，突出抓好就业示范行政村、就业社区、农民工市民化服务中心和青年创业园建设。新增就业岗位4.5万个，转移农村劳动力50万人次。强化社会保障救助。继续扩大社会保险覆盖面。整合城乡居民基本医保，同步推行“六统一”。实施最低生活保障支持计划，进一步提高城乡低保标准。深入推进政府购买居家养老服务。完成市社会福利院扩建一期工程。实现“春蕾助学”行动适龄贫困女童全覆盖。困难残疾人生活补贴和重度残疾人护理补贴做到应发尽发。优化人居生活环境。启动江南、江北垃圾处理场渗滤液处理设备提标改造项目，扎实推进餐厨垃圾及污泥资源化综合利用等项目建设。实施城东热源厂搬迁工程，推进热电厂、自备电站和供热锅炉脱硫脱硝改造，加快淘汰燃煤小锅炉。实施江南污水处理厂扩建和石化园区污水处理厂建设工程。完成哈达山水库水质自动监测站和江北净水厂哈达山取水工程。积极推进宁江区和前郭县殡仪馆易地新建项目。加强城市便民服务。启动城市“一卡通”工程。改造城区背街巷路7条。新建道路中央隔离护栏1.5万延长米。增设交通视频监控点位400个。规划建设一批便民服务广告牌。新建环保公厕100个，实现中心城区和国省级开发区全覆盖。优先发展科技教育。筹划建设市科技馆。深入实施教育“九大工程”。全力推动义务教育均衡发展，全面通过国家义务教育县域基本均衡发展达标验收。支持普通高中解决“办学难”问题。全力争取吉林大学应用技术学院落户松原。启动市实验高中图书科技楼和新教学楼、市中专实训楼、宁江区九年一贯制学校、市青少年科技活动中心等项目建设，完成市逸夫小学教学楼重建和市青少年综合服务平台项目，新建儿童之家30所。加强校园周边环境整治和交通秩序管理。积极发展卫生事业。推动市中心医院易地新建项目建成使用，开工建设市妇幼保健院综合楼易地新建项目，扎实推进全民免费健康体检一期、贫困妇女“两癌”筛查救助、基因检测和优生优育中心项目，扎实做好疾病防控、慢性病治疗、计划生育人口服务等工作。大力发展文化体育事业。实施文体惠民和政府购买文化精品服务工程，送演出下基层335场、电影下乡1.3万场。深入实施全民健身计划，加强社区居民活动场所建设，推动市冰上运动中心项目建成投入使用，办好吉林省第七届少数民族传统体育运动会等重大赛事。

　　（七）聚焦精准抓脱贫。致力于“一道迈入全面小康社会”，科学制定精准扶贫计划，切实加强组织领导，全面落实包保责任，确保55个贫困村21800户3.3万名贫困群众年内脱贫。抓紧抓实脱贫产业。组建扶贫开发公司，发挥其贷款担保等作用，大力发展生猪养殖业和特色种植业，引导农户扩大油莎豆和中药材种植规模，积极推进农村秸秆集中收储和综合开发利用，推动光伏发电、光伏+种植、光伏+养殖等项目落地建设，完成扶贫大数据平台建设等一批脱贫项目，增强贫困村和贫困人口自我“造血功能”。全力补齐设施短板。加快建设乡与村、村与村的连接道路，全面升级供电网络和老旧电力设施，实施贫困村安全饮水、道路硬化、村屯绿化等专项行动，推动光纤和宽带向贫困地区延伸、实现4G网络全覆盖。统筹推进教育医疗等事业发展。给予贫困村教育经费、师资力量、办学条件等更多倾斜，研究建立贫困人口大病救助平台和分类救治大病患者机制，广泛开展就业技能培训，促进剩余劳动力输出转移。

　　（八）维护稳定促和谐。进一步提高社会管理科学化和法治化水平，努力建设平安松原、和谐松原。强化社会综合治理。大力推进七五普法，扩大困难群体法律援助范围。依法加强宗教管理，坚决维护民族团结。强化信访源头治理，努力将矛盾纠纷化解在基层和萌芽状态，依法处理涉法涉诉信访问题，切实解决好群众合理合法的利益诉求。强化安全生产治理。严格落实安全生产责任“五级五覆盖”和企业“五落实五到位”规定，强化源头管控和安全监管执法，坚决遏制重特大安全生产事故发生。强化公共安全治理。严厉打击电信网络诈骗等各类违法犯罪活动，坚决保障人民群众生命财产安全。建成市食品药品检验中心，加强食品、农产品、水产品等检验检测，坚决惩处掺杂使假等危害食品药品安全行为，确保人民群众“舌尖上的安全”。进一步完善公共安全、地质气象等预警体系，开工建设市应急（反恐）指挥中心，完成市规划区震害预测和防震减灾科普基地项目建设，不断提高突发事件综合处置能力。

　　三、加强政府自身建设

　　发展的重任、竞争的态势、人民的期盼，对政府建设提出了新的更高要求。我们要以锲而不舍、驰而不息的精神，长抓不懈，久久为功，扎实推进政府自身建设，努力打造人民满意政府。

　　（一）紧扣依法行政，努力建设法治型政府。全面落实《法治政府建设实施纲要》，建立健全政府依法行政工作报告制度。主动接受市人大及其常委会的法律监督、工作监督和市政协的民主监督，认真执行人大决议决定，定期向人大、政协报告工作。坚持面复制度，认真办理人大代表建议和政协提案。健全法律咨询、公众参与、专家论证、风险评估等制度，坚持重大事项集体讨论决定，确保科学民主决策。严格规范行政执法，认真落实行政执法责任制和行政处罚自由裁量权制度。强化行政执法监督，重点解决执法不规范、不文明等突出问题。建立初任公务员法律培训、测试和行政机关工作人员学法用法制度。

　　（二）紧扣责任担当，努力建设服务型政府。“持之以恒进行政府自我革命”，深入推进“放管服”改革，做好国家和省下放事项承接、落实和后续监管工作。全面推行权力清单、责任清单、负面清单制度并向社会公开。继续深化“五证合一、一照一码”登记制度改革，切实减轻企业和群众负担。全面清理行政审批事项和行政审批中介服务，进一步压缩审批流程。规范并减少行政事业性收费，建立收费项目对外公示制度。全力推进政务公开和政务服务“一张网”建设，努力打造透明、规范、高效、便捷的“互联网+政务”服务体系。

　　（三）紧扣作风转变，努力建设效能型政府。狠抓作风转变和工作落实，加大治庸、治懒、治散力度，力促广大干部真正把精力放在干事业上、把本领用在促发展上。坚决反对形式主义、官僚主义和文山会海，把更多时间和精力用在接地气、抓项目、跑招商上。坚持“一张蓝图绘到底、一张表格考到底”，加强量化考核和过程控制，实施“会诊式”督查，推动任务、责任、进度、绩效“四落实”，努力形成广大干部想干事、能干事、会干事、干成事的生动局面。

　　（四）紧扣从严治政，努力建设廉洁型政府。积极化解政府性债务风险，严控“三公”经费支出，厉行勤俭节约，反对铺张浪费。不断强化行政监察、效能监察和审计监督。严肃查处各类涉软案件，坚决向办事靠讲人情、托关系的不良风气“说不”，强力推进“信用松原”建设，构建亲清新型政商关系，努力把松原打造成为机制更活、信誉更好、环境更优的投资热土，厚植松原发展新优势。深入贯彻落实中央“八项规定”精神和国务院“约法三章”要求，强化纪律和规矩约束，严格落实“两个责任”，全面做好党风廉政建设和反腐败工作，努力营造风清气正的政务环境。

　　各位代表！机遇承载使命，责任考验担当。振兴崛起的艰巨任务，要求我们迎难而上、砥砺奋进；全市人民的信任期盼，鞭策我们披荆斩棘、勇往直前！让我们紧密团结在以习近平同志为核心的党中央周围，在省委、省政府和市委的正确领导下，团结带领全市各族人民，一心为公、勤政为民，攻坚克难、真抓实干，解放思想、改革创新，开拓进取、奋发有为，实现新一届政府良好开局，为“生态振兴、转型崛起”、全面建成“两个城市”、让全市人民生活更加美好而努力奋斗，以优异成绩迎接党的十九大胜利召开！
